# A survey on the perceptions of midwives, women, and support persons on the introduction of a support person information resource

**DOI:** 10.18332/ejm/191162

**Published:** 2024-08-22

**Authors:** Virginia Stulz, Dorothy Dunham, Tara Farrugia, Nicola Drayton

**Affiliations:** 1 Faculty of Health, University of Canberra, Canberra, Australia; 2 Practice Development Unit, Nursing and Midwifery Directorate, Nepean Blue Mountains Local Health District, New South Wales Government, Kingswood, Australia

**Keywords:** birth, women, midwife, support, information resource, support person

## Abstract

**INTRODUCTION:**

Midwives in an Australian birth unit undertook a project to develop a resource for women and their support person. The aim of this study was to explore how the women, support persons and midwives viewed the introduction of this resource designed to guide and support women in their choice of support person.

**METHODS:**

A quantitative survey study was used to explore how three participant groups viewed the introduction of a support person information resource. A hospital designed survey was developed for women, support people and midwives. Data were analyzed using SPSS, version 26 and Braun and Clarke’s guide for thematic analysis.

**RESULTS:**

More than half (55%) of the midwives believed that the information resource presented influenced women’s choice of support people during labor. Almost three-quarters (72%) of the women did not change their choice of number of support people that they wanted during their labor. The majority (83%) of women would recommend the support person brochure to other women. The majority (83%) of support people stayed the entire duration of labor. Four themes were generated from open-ended questions: value of the information sheet, knowing how to be a support person, connecting midwives with being woman-centered, and choosing the support person.

**CONCLUSIONS:**

The availability of an information resource was of benefit for women, support people and midwives, contributing to women feeling more informed in choosing their support person. Midwives felt they had evidence to support conversations with women, contributing to the feeling of being woman-centered. Support people had increased confidence.

## INTRODUCTION

Midwives in the birth unit of a tertiary teaching hospital in the state of New South Wales, Australia, were involved in a program designed to improve the healthcare experiences for all users of the service and healthcare professionals^1^. Midwives in Australia are health practitioners registered with the Australian Health Practitioners’ Regulation Agency^2^, which partners with the National Boards to ensure that Australia’s registered health practitioners are suitably trained, qualified, and safe to practice. Midwives in Australia can work in various areas such as: a public or private hospital, in a caseload model of care or midwifery group practice, and as a privately practising midwife.

The Essentials of Care (EoC) program is underpinned by practice development principles that aim to guide healthcare teams in creating person-centered cultures and generating practice change^3^. The six-phased framework guides teams through a process of exploring and critically thinking about what occurs in practice. This is followed by identifying changes to be made, implementing those changes, and evaluating outcomes.

Midwives have raised concerns about the number of support people and visitors in the birth space. This creates difficulties at times in identifying the key support person and access to the birthing woman. Some women also have expressed feelings of being overwhelmed at such an intimate time and unsure who to have with them, with some being fearful of not hurting family members’ feelings. Exploratory sessions with midwives have identified feelings of frustration with providing a calm and quiet environment, and challenges in knowing who the identified support person was, ways to streamline communication and not repeat the same thing to multiple family and friends. This has impacted the decision-making process women used to choose their support person. Therefore, this identified the need to develop a communication resource that would empower women to choose support people that would enhance their labor and birth experience.

Midwives in the birth unit used this program to gain insight and bring about change to how women are supported in their choice of support person. Twelve months of program engagement resulted in the development of information resources designed to guide women in choosing their support person and information on the role of the support person. The information resources consisted of a support person information sheet (Supplementary file Part 1), provided for women in multiple settings across the health district, and an information poster (Supplementary file Part 2), placed in strategic places across the health district. The resources were available in English, easily read by an 18-year-old, not available online, and contained photos and images. Nine months following the introduction of the information resources, this study was undertaken to explore the perceptions of women, support people, and midwives regarding the potential influence of the resource and how it influenced the women’s choices.

An African study found that information resources appeared to improve pregnant women’s knowledge during pregnancy and provided information on when to report to the health professional if problems were encountered. The information resources were attached to supplement health education given to pregnant women in order to improve perinatal outcomes^4^ Alternatively, one randomized controlled trial showed that leaflets did not change the number of women who reported exercising informed choice in maternity care^5^.

Continuous presence from a support person is shown to correlate with higher rates of vaginal births, reduced analgesia during labor, decreased anxiety, reduced postpartum depression, and overall positive experiences for women^6-8^. A study by Declercq et al.^9^ found the husband/partner provided more than three-quarters (77%) of support during labor, midwifery staff provided almost half (46%) of support, friends or other family members provided almost two-fifths (37%) of support, doctors provided almost a third (31%) of support, and doulas provided 6% of support. Women’s choice in their support person for labor and birth may vary across cultures. In Australia, women are free to choose their support person; however, this is still dependent on various influences within family cultures^9^. Overall, what is clear is that the presence of a support person has a positive impact on women’s birth experience^10,11^.

Midwives have a significant role in developing rapport and supporting the woman and the support person. Midwife involvement has significantly impacted the support person’s confidence and the woman’s birth experience^3,11^. Studies report a lack of knowledge of providing support during labor, which can result in feelings of lack of involvement and anxiousness, resulting in decreased ongoing support in the postpartum period^3,12^.

Key predictors of a positive labor and birth experience are the quality and level of support women receive^13^. Developing resources that can provide information on how to guide and support women in choosing the support person and the role of the support person, can potentially contribute to a positive birth experience. Therefore, the aim of our study was to explore through the application of a survey, how women, support persons, and midwives viewed the introduction of a resource designed to guide and support women in their choice of support person.

## METHODS

### Setting and participants

This study was conducted across maternity services at a tertiary teaching hospital in the state of New South Wales, Australia. The hospital provides services to 4000 births per year. Participants included 41 birthing women, 37 support people, and 42 midwives.

### Design

A survey design was used to explore and understand the perceptions of women, support persons, and midwives regarding introducing a developed support person resource. A survey design was chosen as it offers healthcare researchers a way to gain insight into a particular experience or aspect of health that they can see relates to their clinical practice^14^, thus contributing to understanding the ‘how’ and ‘why’ those being studied experience a particular phenomenon^14,15^. In this study, this was achieved through quantitative and qualitative open-ended questions. Separate surveys were designed for the women, support persons, and midwives. This was necessary in developing an understanding of how the information resource was useful for each participant group^14^. The findings needed to be comprehensible and translated into midwifery practice, aligning with the original aims of the EoC program. The ability to share the findings with midwives was essential to the research. Ethical approval was obtained from the Human Research Ethics Committee for the Local Health District in which the study was conducted 19-31 (A).

### Data collection

Three quantitative surveys were developed for the women, support persons, and for the midwives. Each survey had a combination of open and closed-response questions. The key topics for the open-ended questions in the midwife survey aimed to provide further insight on: 1) what the midwives had noticed since the introduction of the support person resource; 2) the discussions they had with the women; 3) how they used the support person resource in their care of women; and 4) any changes they had noticed in the support person role. The key topics for the open-ended questions in the support person survey were: 1) how the support person resource influenced their role; 2) the types of comfort and support they provided during labor and birth; and 3) how the midwife provided support. The key topics for the open-ended questions in the women’s survey were: 1) any changes they made in relation to their support person; and 2) types of comfort measures their support person provided.

As no pre-validated tool could be found in the literature, each survey was developed by the research team. These surveys were tested with each participant group prior to distribution, and their feedback was used in the final development of each survey. Additionally, the surveys were shared with the local health district consumer group for feedback. The original survey consisted of six questions:

1. How many support people did you have during your labor/birth?
2. Did you find your support people helpful, please describe?
3. We are looking at limiting the number of support people during labor and birth; how would that affect you?
4. On reflection, how many support people would you have preferred during your labor/birth?
5. Did you have visitors whilst you were in the birth unit? How did that work for you?
6. We are looking at limiting the visitors in the birth unit to only the immediate family. How will that affect you?

The survey questions we used in this study are given in Tables 1–3.

**Table 1:** Survey responses from midwives about the support person initiative, Birth Unit, NSW, Australia, 2019 (N=42)

*Questions*	*Responses*	*Number of midwives n (%)*	*Total number of midwives*
1. Do you feel the information on ‘Choosing Your Support Person’ has influenced the choice of whom women choose to have as their support person during labor?	Yes	23 (55)	42
	Unsure	15 (36)	
	No	4 (9)	
2. Have you used the information sheet/poster to start a conversation with women about the choice and role of their support person?	Yes	19 (45)	42
	No	23 (55)	
3. What would you say is the most common number of support people women have with them during labor?	1	1 (2.5)	40
	2	5 (12.5)	
	3	5 (12.5)	
	1–2	1 (2.5)	
	1–3	4 (10)	
	2–3	20 (50)	
	2–4	2 (5)	
	3–4	1 (2.5)	
	5	1 (2.5)	
4. Have you noticed any change in this number of support person/s since the introduction of the information sheet/poster?	Yes	23 (56)	41
	Unsure	13 (32)	
	No	5 (12)	
5. Are you still providing education to women about choosing and the role of the support person?	Always	13 (31)	42
	Most of the time	10 (24)	
	Some of the time	18 (43)	
	Never	1 (2)	
6. Were the women and support person/s aware of skin-to-skin contact during the first hours of the baby’s birth?	Yes	36 (88)	41
	Unsure	5 (12)	
	No	0 (0)	
7. How well do you feel the support person knew and understood the wishes of the woman, please rank on a scale of 0–10, with 0 being the least and 10 being the most. Mean (SD), Range	6.59 (1.67), 3–10		41

**Table 2:** Survey responses from women about the support person initiative, Post-Natal Unit, NSW, Australia, 2019 (N=41)

*Questions*	*Responses*	*Number of women n (%)*	*Total number of women*
1. Prior to the birth of your baby/s, did you know the number of support person/s you wanted with you at the birth of your baby/s?	1	17 (41)	41
	2	17 (41)	
	3	3 (7)	
	1–2	2 (5)	
	2–3	1 (3)	
	No	1 (3)	
2. At the actual birth of your baby/s what was the number of support person/s you had with you at the birth of your baby/s?	0	2 (5)	41
	1	22 (53)	
	2	11 (27)	
	3	5 (12)	
	5	1 (3)	
3. Did you receive the information sheet or notice any posters in our maternity services on ‘Choosing Your Support Person’?	Yes	28 (70)	40
	No	12 (30)	
4. Did the midwife explain the purpose of the information sheet?	Yes	20 (72)	28
	Unsure	4 (14)	
	No	4 (14)	
5. Did the information on ‘Choosing Your Support Person’ encourage you to change the choice of whom you had with you during the birth of your baby/s?	Yes	8 (28)	29
	No	21 (72)	
6. Would you recommend the ‘Choosing Your Support Person’ information to other women?	Yes	24 (83)	29
	Unsure	5 (17)	
	No	0 (0)	
7. Did your support person/s stay with you during your entire duration of labor?	All of the time	33 (82.5)	40
	Most of the time	6 (15)	
	Some of the time	1 (2.5)	
	Minimal time	0 (0)	
8. Did you and your baby share uninterrupted skin-to-skin contact immediately following birth?	Yes	33 (82.5)	40
	Unsure	1 (2.5)	
	No	6 (15)	
If yes, how long did it last?	10 min	3 (9)	32
	15 min	2 (6)	
	20 min	2 (6)	
	30 min	3 (9)	
	45 min	1 (3)	
	1 h	8 (25)	
	1 h 30 min	1 (3)	
	1 h 45 min	1 (3)	
	2 h	8 (25)	
	5 h	1 (3)	
	8 h	1 (3)	
	2 days	1 (3)	
9. How long after your birth did you have visitors?	1 h	10 (25)	40
	2 h	8 (20)	
	3–8 h	8 (20)	
	9–24 h	12 (30)	
	24–48 h	2 (5)	
10. How helpful was the ‘Choosing Your Support Person’ information in guiding you on choosing your support person, please rate on a scale 0–10, with 0 being least helpful and 10 being the most helpful. Mean (SD), Range	8.14 (2.12), 2–10		28

**Table 3:** Survey responses from support people about the support person initiative, Post-Natal Unit, NSW, Australia (N=37)

*Questions*	*Responses*	*Number of support people n (%)*	*Total number of support people*
1. Did you receive the information sheet or notice any posters in our maternity services on ‘Choosing Your Support Person’?	Yes	20 (55)	36
	Unsure	6 (17)	
	No	10 (28)	
2. Did the information on the sheet or posters help guide you in providing support for your wife, partner, relative, or friend?	Yes	19 (53)	36
	Unsure	8 (22)	
	No	1 (3)	
	Did not read or receive information	8 (22)	
3. Were any of the comfort measures taken from suggestions in the ‘Choosing Your Support Person’ information?	Yes	15 (44)	34
	No	5 (15)	
	Did not read or receive information	14 (41)	
4. Were you the only support person?	Yes	19 (51)	37
	No	18 (49)	
5. How long did you stay with your wife, partner, relative, or friend during their labor?	All of the time	28 (80)	35
	Most of the time	5 (14)	
	Some of the time	2 (6)	
	Minimal time	0 (0)	
6. Was uninterrupted skin-to-skin contact between mother and baby shared immediately following birth?	Yes	30 (83)	36
	Unsure	0 (0)	
	No	6 (17)	
If yes, how long did it last?	10 min	2 (8.25)	24
	15 min	2 (8.25)	
	20 min	1 (4)	
	30 min	2 (8.25)	
	1 h	10 (42)	
	1 h 30 min	2 (8.25)	
	2 h	4 (17)	
	3 h	1 (4)	
7. Would you recommend the information to other people that are providing support during birth?	Yes	27 (75)	36
	Unsure	0 (0)	
	No	0 (0)	
	Did not read or receive information	9 (25)	
8. If you read the information, how helpful was it, please rate on a scale 0–10, with 0 being least helpful and 10 being the most helpful. Mean (SD), Range	7.85 (1.8), 5–10		20

The survey was distributed nine months after the information resource was first introduced. The survey remained open for six months in 2019. Women and support persons were invited to complete the survey by midwives close to discharge from the post-natal unit. They were informed the surveys were voluntary and anonymous, and a confidential closed return box was placed on the postnatal unit for completed surveys. Midwives from the birth unit were invited to complete the study via the birth unit’s internal mail system. They also had a confidential closed return box to return surveys.

### Data analysis

Survey data were analyzed descriptively using frequencies and percentages, and chi-squared analyses to determine associations and relationships between the questions in Statistical Package for Social Science (SPSS) version 26.

The Braun and Clarke^16^ framework for thematic analysis was used for the analysis of the open-ended questions. The open-ended comments from each survey were transcribed verbatim and analyzed for themes.

The generation of themes supports the study’s objective of discovering the perceptions of midwives, women, and support people. Themes were generated by undertaking a process of reading and re-reading the data by the researchers, identifying and labelling codes in the data, and developing themes^16^. Regular meetings were held with the researchers to revisit the data until a consensus was reached. The trustworthiness of the data was established by the researcher’s continued re-immersion with the data until agreement that the generated themes were a true reflection of the data^17^.

## RESULTS

### Survey results

*Midwife survey*

Forty-two midwives completed the survey (Table 1). More than half (55%) of the midwives believed that the information resource presented influenced women’s choice of support people during labor, and more than half (56%) noticed a change in the number of support people for women following the introduction of the resource. The midwives reported that the majority (88%) of women and support people were aware of skin-to-skin contact for the first few hours of the baby’s birth. The most common forms of support that the midwives noticed that the support person provided were providing drinks (22%), ice (20%), a cool face cloth (15%), and massages (14%). Midwives were six times more likely to notice if they had a conversation with women using the information resource and that this influenced the change in number of support people for the women [χ^2^(1)=12.85, p<0.005].

*Women survey*

Forty-one women completed the survey, although almost three-quarters (72%) of women did not change their choice of the number of support people that they wanted during their labor. The majority (83%) of women would recommend the support person brochure to other women (Table 2). The most common forms of support that the support person provided were providing drinks (19%) and ice (18%), followed by repositioning (15%) and massages (14%). The majority (83%) of support people stayed the entire duration of labor, and the majority (83%) of women shared skin-to-skin contact with their baby in the first few hours of birth.

*Support person survey*

Thirty-seven support people completed the survey (Table 3). The most common forms of support that the support person provided were providing drinks (20%), ice (17%), repositioning (15%), and massages (15%), in agreement with the midwives’ responses. More than half (53%) of the support people reported that the information resource helped and guided them in being a support person for their loved ones during labor. The four themes generated from the open-ended questions revealed the perceptions of women, support people, and midwives. These themes included: 1) The value of the information sheet, 2) Knowing how to be a support person, 3) Connecting midwives with being woman-centered, and 4) Choosing the support person.

*Value of the information sheet*

The information resource provided women with the knowledge to think more about who they would like as a support person. They felt more confident in making their choices and supported by the information provided in the resources and conversations with the midwives. Support persons felt the information resource gave them knowledge on who the support person could be, the types of things to do, what to bring to the hospital, and what happens after discharge. This led to support people feeling more confident in their ability to provide comfort and support.

Below are relevant quotes from the interviews. The quotes of midwives are coded as M, for women as W, and for support persons as SP:

*‘I knew how to support my wife during labor, how to handle the baby afterwards and create comfort for my wife.’ (SP11) ‘It provided me with the understanding of what a support person is there for.’* (W4)

*‘Being a supportive husband, the information I read made the experience easier and less stressful.’* (SP8)

*‘It told me who can be the support person, what a support person needs to do, what to bring to hospital and what the process is.’* (SP3)

*Knowing how to be a support person*

Support people expressed feeling knowledgeable about what to do to support their loved ones during labor. In addition to the resource, they had conversations with the midwives and learned how to assist with different positions in labor, how to use a peanut ball, shower, and other comfort measures. This led to feeling more confident in their ability to provide comfort and support. The midwives noticed that support people were advocating for the women and that they tended to be more active in their role during birth:

*‘They are more willing to advocate and more proactive in asking for heat/cool packs, initiating massage, and applying cool cloths.’* (M15)

*‘I have noticed support people will act on the information that will benefit the women.’* (M4)

*‘I had suggestions on ways to push, advice on using the peanut ball and shower to ease discomfort.’* (SP7)

*‘I learned how to give reassurance, positive messages and hypnobirthing terminology and techniques.’* (SP21)

*Connecting midwives with being woman-centered*

The midwives described being woman-centered in their ability to have conversations with women about their choice of support person and developing a birth plan. Woman-centered care is a fundamental feature of midwifery practice borne from the professional philosophy of being ‘with woman’^14^. Midwives noticed more birth plans were being developed, which was significant as they felt this was directly related to providing woman-centered care. Having the resource provided additional evidence to support their conversations with women. Midwives felt the resource encouraged greater capacity for shared decision-making as the resource provided a guide to talk about the upcoming birth and women’s wishes. During these conversations, midwives felt they could guide the woman in identifying the support person who would best contribute to a positive birth experience:

*‘Women that have received the information in ante-natal come with prior knowledge about their choice of support person, this gives us an opportunity to emphasize the importance that the choice is theirs.’* (M8)

*‘What I notice is that support people will act on the information provided, I feel that the information is giving midwives the opportunity to discuss what is of benefit to the woman.’* (M24)

*‘I get to discuss the need to be careful when selecting support people on who will be calm, supporting and encouraging during labor.’* (M38)

*‘I notice more birth plans are being developed which makes me feel that woman-centered care is at the forefront of our minds, this allows me to discuss what we believe as a birth unit is best for how we can support the woman.’* (M24)

*‘It is the woman’s birth experience, not ours, it’s reminder for us to relax about how many support people there are’; the women can decide who they want, that is woman-centered care.’* (M33)

*‘This is definitely a step forward to woman-centered care, having the information resource together with the midwife gives a chance to talk about how the woman and support person can add to the experience.’* (M12)

*Choosing the support person*

Women felt informed about choosing their support person, they described changing the choice of their support person because of reading the information. They had a greater understanding of the role of the support person, which assisted them in making an informed choice. In some instances, midwives noticed that women had a greater number of support people. However, most midwives felt the number of support people were reduced, and women had a clearer understanding of who they wanted at birth. Midwives expressed not needing to explain the role of the support person during birth, and that support people were more actively involved in supporting the woman with her birth plan:

*‘I had originally chosen my sister to support me, then changed to my mum.’* (W9)

*‘I ended up asking my husband to be my support person instead of my sister.’* (W6)

*‘I have noticed women started to think who they really wanted as their support person in labor, rather than just accepting whoever is present at birth.’* (M9)

*‘The information was sufficient and detailed; it helped me to know everything I needed in my support person.’* (W15)

## DISCUSSION

The findings from this study demonstrate the value of having an information resource in enhancing women and supporting a person’s birth experience. Midwives found value in the resource, as it was not only the information resource that made a difference to women, but it established a platform for them to engage in conversations with the women and their support person. An important part of a midwife’s role is to discuss the woman’s choice of whom she wants at her labor and birth, and perhaps this was not happening so often and why this information resource was an important initiation.

Midwives confirmed that the relationship between women and the midwife extends beyond them to include women’s support people and significant others^18^. Having the availability of information may benefit midwives in having conversations surrounding the woman’s wishes in birth and labor. Similarly, one study that evaluated sources of information accessed by pregnant women to meet their information needs, showed that discussion with a midwife was the most frequently used source of information^19^.

The opportunity to get to know a woman and her hopes for her labor and birth experience enhances communication between the woman and midwife^11,18^. Focusing more on women’s choices was a reminder of the importance of woman-centered care^20^. Exploring ways a midwife can feel connected in providing woman-centered care is an important aspect of what it means to be a midwife. Developing and evaluating these opportunities is important in contributing to the midwifery profession.

The information resource influenced the number of birth plans being developed by women, with midwives noticing women were more proactive in their labor and birth choices. Previous research has shown that women who had written birth plans were less likely to experience interventions such as augmentation with oxytocin, artificial rupture of membranes, and less likely to have an epidural^21^. Writing the birth plan encourages open discussion between the midwife and women surrounding her options, aligning with woman-centered care^22^.

Support people tended to be more proactive in asking for supportive measures during birth and showed a greater understanding of providing support. This is consistent with other studies that have found that fathers were 1.06 times more likely to be involved in the birth experience if they had received prior information^23^. Other research has shown that if partners are provided with professional support for supporting women during childbirth, this increases the ability to provide individualized care^12^.

The information resource provided women with more insight of whom they could have as their support person and the types of support they could receive. Women have reported gratitude from their partners in providing support with practical things such as a cool face wash and sips of water^24^, and this correlates with this study’s findings which showed support people provided drinks, ice, and cool face cloths. Women in this study would recommend this information resource to other women, further highlighting its importance. Support people also reported staying with the woman for the duration of labor. The continuous support by women’s support people during labor can increase the birthing women’s satisfaction because of understanding the women’s needs and wishes^24-26^.

The birth setting, culture, and personal relationships can influence women’s support choices. Quite often, support people fulfill a vital role that midwives may not always be able to provide^27^. The COVID-19 pandemic placed further restrictions on women’s support people during labor, being restricted to one person, and often women had to make difficult decisions to exclude their partners or other children during their labor. Midwives found that without extra support, they engaged more with other women and got more involved with women during labor^28^. Our study showed that midwives felt that if they discussed the women’s birth plans, women were more likely to be more informed about the role of the support person and to change the number of support people. However, the women did not report these results in their survey, with almost three-quarters reporting that they did not change their support people. Similarly, one qualitative study identified that information resources developed for pregnant women did not promote informed choice^29^.

The World Health Organization (WHO) and UNICEF^30^ recommend skin-to-skin contact as soon as the mother is alert and safe for the mother and the baby. Skin-to-skin is placing the naked newborn prone on the mother’s bare chest after birth and covering the baby with a warmed blanket^31^. This study provides good evidence from the midwives, women, and support people that most women experience skin-to-skin contact immediately following birth. Skin-to-skin promotes optimal immunological, physiological, and cognitive effects on the neonate, resulting in early initiation of breastfeeding with increased breastfeeding duration and maternal confidence and bonding^32-34^.

Further study is recommended on the types of resources that contribute to a positive birth experience, enhance the role of the support person, and enable midwives to feel they are providing woman-centered care.

### Limitations

Participants were only recruited from one tertiary teaching hospital within a Local Health district in New South Wales. This could limit the transferability of the findings and is a limitation of this study. This study was initiated prior to the impact of COVID-19 and may not necessarily reflect the restrictions on support for women during birth and labor during the pandemic.

## CONCLUSIONS

The findings from this study provide evidence that women want to be informed about how to choose a support person. Having an information resource that provided a platform to further engage in conversations with the midwife to identify the support and influence a positive birth experience. Although three-quarters of women did not change the number of support people, it contributes to the importance of support personnel at birth. This study has important limitations due to the pandemic intervention in its development. The resource contributed to midwives feeling that they were providing women-centered care through shared decision-making and supporting women in their choices. Support people had improved knowledge of their role and needed less guidance from the midwife during birth and labor. One of the greatest contributions was that the resource enhanced and enabled conversations between the midwife, women, and support person surrounding their labor and birth, resulting in a positive birth experience.

## Supplementary Material



## Data Availability

The data supporting this research can be found in the Supplementary file.
