# Supplementary material for: A survey on the perceptions of midwives, women, and support persons on the introduction of a support person information resource
Source: Eur J Midwifery. 2024 Aug 22;8:10.18332/ejm/191162. doi: 10.18332/ejm/191162 (PMC11339884; doi:10.18332/ejm/191162)
Supplement: Supplementary file 1 [file EJM-8-45-s1.pdf]

Choosing your SUPPORT person

Your labour and the birth of your baby is a very personal and special time for you

Although you will be supported by a midwife throughout labour, it is important to have another support person with you. Usually your partner or another person you are close to. One to three people is an optimal number.

Choose support people who you will feel comfortable with who can provide you with continuous reassurance, encouragement and guidance.

After your baby is born

Skin to skin contact in the early hours after birth helps babies to make an easier adjustment to life outside of the womb.

The staff are aware that family and friends are keen to welcome the new baby however there are important things that need to be done before visitors arrive.

Let your family and friends know you may need some quiet time immediately after the birth of your baby. This precious time is for you to get to know your baby.

Your support person can assist you by:

- Creating a quiet, calm environment - dimming the lights, having relaxing music playing and limiting disruptions
- Providing physical comforts like ice, water, massage or heat packs. Support you in upright and active positions
- Offering emotional support and positive encouragement to help you remain calm and focused during your labour and birth
- Being a communicator by taking phone calls and passing on messages to family and friends

NSW GOVERNMENT
Health
Nepean Blue Mountains
Local Health District

INFORMATION SHEET

Birthing is one of the most intimate, instinctive and personal experiences of a woman's life

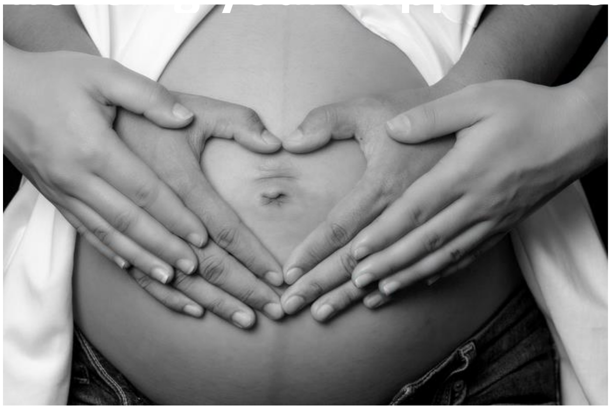

Research has shown that women who have continuous emotional and physical support during labour are less likely to need pain relief and will have a shorter labour and better birth outcomes.

Your labour and the birth of your baby is a very personal and special time for you.

Although you will be supported by a midwife throughout your labour, it is important to have another support person with you. Usually your partner or another person you are close to.

One to three people is an optimal number.

Choose support people who you will feel comfortable with and who will help you rather than distract you during the different stages of labour.

We encourage your support people to stay with you during your labour and the birth of your baby to provide continuous support.

If you have too many people with you, it may affect your ability to cope with the progress of your labour.

TIS 131450

By creating a quiet, calm and safe space to birth, support people can provide you with reassurance, encouragement and guidance.

This boosts the right hormones for natural birth, which reduces the need for interventions that could cause separation from your baby.

You and your support people can prepare for labour and birth by attending antenatal classes, reading reliable information and talking to the healthcare professionals who are caring for you.

Your support person can assist you by:

- Creating a relaxing environment by dimming the lights and have music playing. The hormones needed for birth are increased by having a space that is warm, quiet, calm and without a lot of disruptions.
- Help to make you physically comfortable by providing you with ice, water, a massage or help with heat packs for your back, place a cool face cloth on your forehead and neck and support you in upright and active positions.

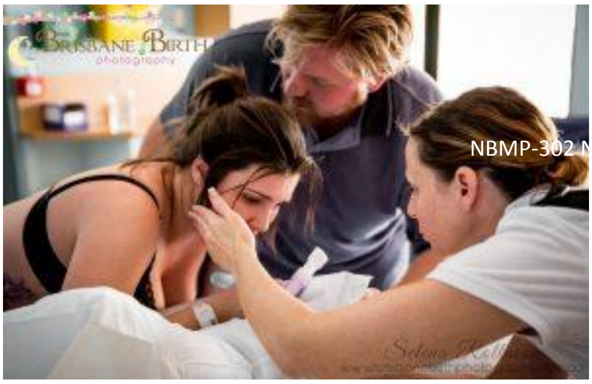

- Provide positive encouragement, your support

person can offer emotional support and reassurance that will help you to remain calm and positive during your labour and birth. Your support person can help by reminding you that each contraction will bring you closer to meeting your baby.

- Being a communicator by taking phone calls and passing messages onto inquiring friends and family.
- Looking after themselves. Your support person should take a break if they need to. They might like to step outside to get a drink and some fresh air.

After your baby is born

The time after your baby is born is a very special time.

We recommend uninterrupted skin-to-skin immediately after birth for you and your baby to bond (baby naked, or in a nappy, placed against the mother's bare chest).This should be for at least the first hour after birth.

Skin to skin contact in the early hours after birth helps babies make an easier adjustment to life outside the womb.

The positive effects on your baby include the following:

- Baby's skin temperature, heart rate and breathing rate are more likely to be stable and within normal range
- Baby's blood glucose levels are more likely to be stable and within normal range.
- Baby is less likely to cry

If you choose to breastfeed, some of the additional benefits are:

- Your baby is more likely to attach and feed well at your breast
- Your baby is more likely to breastfeed exclusively for longer.

Mothers who have skin to skin contact after birth are more likely to feel confident and comfortable in meeting their baby's needs.

Skin to skin contact is good for all babies whether you choose to breast or formula feed.

The staff are aware that family and friends are keen to welcome your new baby however there are important things that need to be done before your visitors arrive.

Let your family and friends know you may need some quiet time immediately after the birth of your baby. This precious time is for you to get to know your baby.

Nepean Hospital Birth Unit
Tel No: 4734 2294

Blue Mountains Hospital Birth Unit
Tel No: 4784 6572

Lithgow Hospital (switchboard)
Tel No: 6350 2300

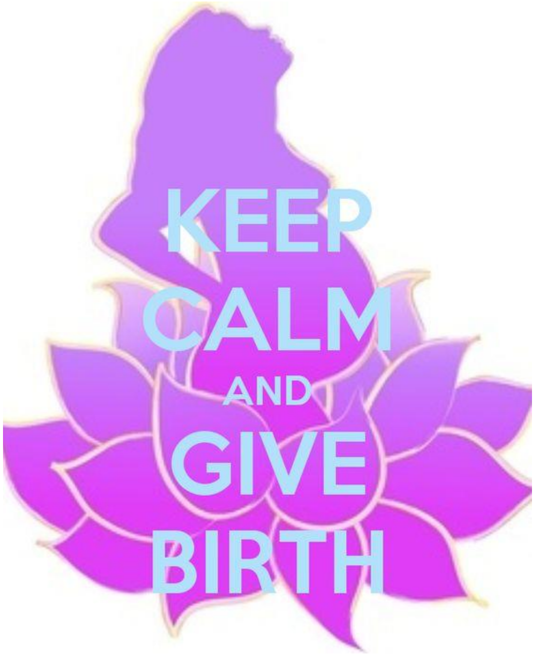

Table S1. Qualitative Research Review Guidelines (RATS) checklist.

Qualitative Research Review Guidelines – RATS [1]		
**Ask this of the manuscript**	**This should be included in the manuscript**	**Where item has been addressed**
**R – Relevance of study design**		
Is research question interesting?<br><br>Is research question relevant to clinical practice, public health, or policy?	Research question explicitly stated<br><br>Research question justified and linked to the existing knowledge base (empirical research, theory, policy)	Question provided on page 2 under the aim.<br><br>Page 2 under introduction
**A – Appropriateness of qualitative method**		
Is qualitative methodology the best approach for the study aims?<br>*Interviews*: experience, perceptions, behavior, practice, process<br>*Focus groups*: group dynamics, convenience, non-sensitive topics<br>*Ethnography*: culture, organizational behavior, interaction<br>*Textual analysis*: documents, art, representations, conversations	Study design described and justified e.g., why was a particular method (i.e., interviews) chosen?	Pages 2 and 3 under design
**T – transparency of procedures**		
Sampling<br><br>Are the participants selected the most appropriate to provide access to the type of knowledge sought by the study?<br><br>Is the sampling strategy appropriate?	Criteria for selecting the study<br><br>sample justified and explained<br>*theoretical*: based on pre conceived or emergent theory<br>*purposive*: diversity of opinion<br>*volunteer*: feasibility, hard-to-reach groups	Page 3 and 4 under setting and participants, design and data collection
Recruitment<br>Was recruitment conducted using appropriate methods?<br><br>Is the sampling strategy appropriate?	Details of how recruitment was conducted and by whom<br><br>Details of who chose not to participate and why	Pages 3 to 4<br><br>Page 3

Could there be selection bias?

Data collection<br>Was collection of data systematic and comprehensive?	Method (s) outlined and examples given (e.g., interview questions)	Page 7, Appendix Figure 1A<br>Page 5 and table 1, 2, 3
Are characteristics of study group and setting clear?	Study group and setting dearly described	Page 3 and 4
Why and when was data collection stopped, and is this reasonable?	End of data collection justified and described	Page 3
Role of researchers<br>Is the researcher(s) appropriate?	Do the researchers occupy dual roles (clinician and researcher)?	Page 4 under data analysis
How might they bias (good and bad) the conduct of the study and results?	Are the ethics of this discussed? Do the researcher(s) critically examine their own influence on the formulation of the research question, data collection, and interpretation?	Page 4 under data analysis
Ethics<br>Was informed consent sought and granted?	Informed consent process explicitly and dearly detailed	Page 4
Were participants'' anonymity and confidentiality ensured?	Anonymity and confidentiality discussed	Page 3 and 4
Was approval from an appropriate ethics committee received?	Ethics approval cited	Page 4
**S - Soundness of interpretive approach**		
Analysis<br>Is the type of analysis appropriate for the type of study?	Analytic approach described in depth and justified	Page 4
Thematic: exploratory, descriptive, hypothesis generating<br>*Framework:* e.g. policy<br>*Constant comparison/grounded theory:* theory generating, analytical	Indicators of quality:<br>Description of how themes were developed from the data (inductive or deductive)<br>Evidence of alternative explanations being sought<br>Analysis and presentation of negative or deviant cases<br>Description of the basis on which quotes were chosen	Page 4
Are the interpretations dearly presented and adequately supported by the evidence?	Semi-quantification when appropriate<br>Illumination of context and/or	Page 5 through to 9 & tables 1, 2, 3
Are quotes used and are these appropriate and effective?	meaning, richly detailed	Page 5 through to 9

Was trustworthiness/reliability of the data and interpretations checked?	Method of reliability check described and justified e.g., was an audit trail, triangulation, or member checking employed?<br><br>Did an independent analyst review data and contest themes? How were disagreements resolved?	Pages 8 through to 10 and Page 4
Discussion and presentation<br>Are findings sufficiently grounded in a theoretical or conceptual framework?<br><br>Is adequate account taken of previous knowledge and how the findings add?<br><br>Are the limitations thoughtfully considered?<br><br>Is the manuscript well written and accessible?	Findings presented with reference to existing theoretical and empirical literature, and how they contribute<br><br>Strengths and limitations explicitly described and discussed<br><br>Evidence following guidelines (format, word count)<br><br>Detail of methods or additional quotes, contained in appendix<br><br>Written for a health sciences audience	Pages 9 through to 11<br><br>Page 11<br><br>Pages 1 through to 12<br><br>Yes, conclusion confirms relevance for midwives
Are red flags present?<br><br>These are common features of ill-conceived or poorly executed qualitative studies, are a cause for concern, and must be viewed critically<br><br>They might be fatal flaws, or they may result from lack of detail or clarity	Grounded theory; not a simple content analysis but a complex, sociological, theory generating approach<br><br>*Jargon*: descriptions that are trite or jargon filled should be viewed skeptically<br><br>*Over interpretation*: interpretation must be grounded in "accounts" and semi-quantified if possible or appropriate<br><br>*Seems anecdotal, self-evident*: may be a superficial analysis, not rooted in conceptual framework or linked to previous knowledge, and lacking depth	-

	Consent process thinly discussed: may not have met ethics requirements<br><br>*Doctor-researcher*: consider the ethical implications for patients and the bias in data collection and interpretation	

[1] The RATS guidelines modified for BioMed Central are copyright Jocalyn Clark, BMJ. They can be found in Clark JP: **How to peer review a qualitative manuscript**. In *Peer Review Health Sciences*. Second edition. Edited by Godlee F, Jefferson T. London: BMJ Books; 2003:219-235

© 2024 Stulz V. et al.
